# Sex-Specific Temporal Trends in Overweight and Obese Among Schoolchildren From 2009 to 2018: An Age Period Cohort Analysis

**DOI:** 10.3389/fped.2021.615483

**Published:** 2021-05-13

**Authors:** Yung-Chieh Chang, Wan-Hua Hsieh, Sen-Fang Huang, Hsinyi Hsiao, Ying-Wei Wang, Chia-Hsiang Chu, Shu-Hui Wen

**Affiliations:** ^1^Department of Pediatrics, Hualien Tzu Chi Hospital, Buddhist Tzu Chi Medical Foundation, Hualien, Taiwan; ^2^Department of Public Health, College of Medicine, Tzu-Chi University, Hualien, Taiwan; ^3^Center for Physical Education Teaching, College of Education and Communication, Tzu Chi University, Hualien, Taiwan; ^4^Department of Social Work, College of Humanities and Social Science, Tzu Chi University, Hualien, Taiwan; ^5^School of Medicine, Tzu Chi University, Hualien, Taiwan; ^6^Department of Family Medicine, Hualien Tzu Chi Hospital, Buddhist Tzu Chi Medical Foundation, Hualien, Taiwan; ^7^Hualien County Health Bureau, Hualien, Taiwan

**Keywords:** age-period-cohort analysis, body mass index, childhood obesity, gender, poisson regression

## Abstract

**Background:** Our study examined the age, period, and cohort effects on overweight and obesity in children using a 10-year dataset collected from schoolchildren in Hualien, Taiwan.

**Methods:** We used data from the annual health checkup of a total of 94,661 schoolchildren in primary schools and junior high schools in Hualien from 2009 to 2018. Children were defined as overweight or obese by the gender- and age-specific norm of the body mass index. We conducted the age-period-cohort (APC) analysis in boys and girls separately.

**Results:** From 2009 to 2018, the rates of children overweight and obese were 12.78 and 14.23%, respectively. Boys had higher rates of overweight and obesity than girls (29.73 vs. 24.03%, *P* < 0.001). Based on APC analysis results, positive age effect existed regardless of gender. The risk of overweight or obesity of children aged 9 or 12 years was significantly higher compared to the average rate. As for period effect, a fluctuating downward trend in overweight was evident in 2016, and a similar trend in obesity was seen in 2017 across gender groups. The birth cohort of 2007 to 2009 had a significant higher proportion of overweight and obese than other birth cohorts. This indicated that the proportion of children overweight and obese in the young generation is higher than that in the old generation.

**Conclusion:** An increased risk of children overweight or obese was associated with age and later birth cohort. For the period effect, the trend in the prevalence of overweight and obesity fluctuated downward slowly from 2016 to 2017.

## Introduction

The prevalence of overweight and obesity among children and adolescents increased globally. The World Health Organization (WHO) estimated that the number of overweight children under the age of 5 years was over 41 million, and over 340 million children and adolescents aged 5–19 years were overweight or obese in 2016. Persisted-early childhood obesity was associated with an increased risk of high blood pressure (1). The risk of early-onset diabetes in children and adolescents was greatly magnified with increasing severity of obesity (2). In the United States, scholars warned that childhood obesity continues to be a significant concern and the levels of severe obesity in all children aged 2–19 years and populations have seen an increase in the past 18 years (3). In Taiwan, an increasing trend in overweight and obesity was observed among Taiwanese children and adolescents in a 2-year period (4). The Nutrition and Health Survey in Taiwan (NAHSIT) 2013–2016 report showed that the prevalence of childhood overweight and obesity among children aged 7–12 years was 28.4%. In Hualien, the overall prevalence of overweight and obesity was 25% in 2010. The prevalence of overweight and obese was also increased with higher grades (5).

Due to the complexity of developing childhood overweight and obesity, Dr. Davison and Birch proposed the Ecological System Theory (EST) in 2001 which highlighted the importance of ecological niches including the family, school, community, and society. Specifically, child characteristics that place children at risk of the development of overweight (including dietary intake, physical activity, and sedentary behavior) should be embedded in this EST model and tested through causal processes (6). Dr. McCrindle defined a birth cohort between 1997 and 2010 as “generation z” and children born between 2010 and 2025 as “generation alpha or i” (7). Apple Inc., released the first generation of iPad in 2010 which placed children after generation z under the convenient environment fulfilled with high technologic services such as internet and social media. Children and adolescents of generation z and alpha were tight up with virtual activities such as online chat, gaming, shopping, and entertainment and became more sedentary in their daily routine.

With changing environment and time period, to our knowledge, few studies explored the multifactorial causations of childhood obesity through age, period, and cohort (APC) analysis which distinguished three types of time-varying phenomena: age, period, and cohort effects. A previous study used data of children and adolescents aged 2–25 from 1989 to 2009 in China and found a U-shape of age effects, positive period effects, and cohort effects in males (8). Another APC analysis of Iranian children and adolescents indicated a decreasing trend of age, positive period effects, and cohort effects (9). Regarding the magnitude of BMI, a study conducted in Hong Kong, China, found that BMI increased with age and period, but a higher BMI was seen in older cohorts (10). In Taiwan, few papers derived through APC analysis in childhood overweight and obesity, with only one APC model of overweight and obesity in Taiwanese adolescents (aged 12–18), found a decreasing trend with age and period, and a positive birth cohort effect (11). However, the effects of age, period, and cohort on the rising temporal trends of children overweight and obesity remain unclear.

The Taiwan Ministry of Health and Welfare and Ministry of Education launched the Health-Promoting School (HPS) program in 2002, and the HPS program gradually expanded to all counties in Taiwan (12). In 2010, the Ministry of Education initiated a program to provide empowerment strategies for training school faculty, staff, and students to participate actively in school health management; linkage to community resources was also disseminated. In light of current evidence and the recent HPS program in Taiwan, it is important to understand the effects age, period, and cohort may have had on the childhood overweight and obesity. In this study, we used a 10-year long-term dataset (2009–2018) of schoolchildren aged 6–12 in Hualien county in Taiwan to investigate the temporal trends of the age, period, and cohort effects in childhood overweight and obesity.

## Materials and Methods

### Study Samples

We recruited a population-based sample of schoolchildren in Hualien county between 2009 and 2018. Hualien, the largest county of Taiwan by area with a population of 350,000, has implemented a physical health checkup program once per year. Students from the first (aged 6 years, termed grade 1), fourth (aged 9 years, termed grade 4), and seventh grades (aged 12 years, termed grade 7) were participated into the program as per the regulations by the Taiwan Ministry of Education. A total of 95,749 students from 107 primary schools and 25 junior high schools were eligible. The annual completion rates ranged from 98.8 to 99.3%. Children without complete data of birth date, height, or weight were excluded (*n* = 1,088). Thus, a total of 94,661 eligible schoolchildren were recruited. The highest proportion of children was in 2009 (12.61%) and declined annually to 8.53% in 2018 because of a decreased population size (Table 1). This study was approved by the Institutional Review Board of Hualien Tzu Chi Hospital in Hualien, Taiwan (REC No.: IRB109-061-B).

**Table 1:** Sample sizes based on age and gender at each examination year (*n* = 94,661).

	**Age, 6 y**		**Age, 9 y**		**Age, 12 y**				
**Period**	**Boys**	**Girls**	**Boys**	**Girls**	**Boys**	**Girls**	**Total sample size**	**Percentage of total (%)**	**Population size**
2009	1,762	1,481	2,025	1,972	2,439	2,261	11,940	12.61	12,055
2010	1,613	1,454	1,981	1,795	2,211	2,017	11,071	11.70	11,202
2011	1,463	1,397	1,710	1,632	2,137	1,909	10,248	10.83	10,361
2012	1,501	1,265	1,715	1,510	2,090	2,000	10,081	10.65	10,200
2013	1,360	1,234	1,570	1,434	2,032	1,852	9,473	10.01	9,565
2014	1,359	1,227	1,481	1,393	1,793	1,709	8,962	9.47	9,021
2015	1,332	1,200	1,463	1,282	1,813	1,585	8,675	9.16	8,733
2016	1,243	1,112	1,359	1,245	1,687	1,533	8,179	8.64	8,236
2017	1,252	1,098	1,347	1,254	1,537	1,470	7,958	8.41	8,030
2018	1,403	1,241	1,339	1,235	1,514	1,342	8,074	8.53	8,133

### Data Measurements

Annual physical examination items included body composition, vision, oral health, and urine test. This study used a part of the data measurements from the physical examination. Study variables were examination year, demographic variables (gender, age, grade, and residential area), and body composition such as height and weight. Age was calculated by subtracting the examination data from the birth date. Height and weight were measured by trained school nurses at school according to the standard procedures and instruments. Body mass index (BMI) was calculated as weight (kg)/height^2^ (m^2^). Child overweight or obesity was defined as BMI between the 85th and 95th percentiles or more than the 95th percentile among children of the same age and sex according to the age–sex-specific BMI cutoff values from the new growth charts for Taiwanese children and adolescents, based on the World Health Organization (WHO) standards, which were developed by the Department of Health in Taiwan in 2010 (13). Residential area was classified into industrial, sub-industrial, and remote area based on level of development and social-economic status (14). The collected dataset in our retrospective cohort study was from the annual health examination records. Previous BMI category and daily dietary and activity records were not available for each schoolchild in our dataset.

### Statistical Analysis

Descriptive statistics were presented for examination year, demographic variables, height, weight, BMI, and weight status by sex group. For comparison of sex group, the independent sample *t*-test and Chi-square test were applied when appropriate. We investigate sex-specific temporal trends of the prevalence of overweight and obesity in terms of three temporal factors: age (6, 9, 12 years old), period (examination year), and cohort (birth year). The prevalence rates of sex-specific overweight and obesity between 2009 and 2018 were modeled by the Poisson log-linear model. All models were analyzed separately for boys and girls. The Poisson log-linear model is commonly used in epidemiology where the counts of events such as overweight or obesity are assumed to follow Poisson distributions and the rates of overweight or obesity are estimated by the log-linear model. The model can be expressed as:

$$\begin{array}{l} \text{log}r_{ij}=\mu +\alpha_{i}+\beta_{j}+\gamma_{k},i=1,\ldots ,a.j=1,\ldots ,p \end{array}$$

where *r*_*ij*_ denotes the expected prevalence of overweight or obesity at the i-th age group and the j-th examination year; μ denotes the overall population mean; α_*i*_ denotes the effect of the i-th age group (*a* = 3); β_*j*_ denotes the effect of the j-th examination year (*p* = 10); and γ_*k*_denotes the effect of the k-th cohort. We estimated the age, period, and cohort effects by using the intrinsic estimator method based on the intention-to-collapse method (15, 16). In other words, because the age groups are 3 years apart, three continuous periods were collapsed into one to have the same time span of age groups. The birth cohorts by the intention-to-collapse method were categorized into the birth years 1997, 1998–2000, 2001–2003, 2004–2006, 2007–2009, and 2010–2012 (Supplementary Table 1). Notice that the intention-to-collapse method does not change the coding of the age and period effects. For an intuitive interpretation, we calculated the rate ratio (RR) as the exponential values of the regression coefficients (17, 18). It means the risk of overweight or obesity at a particular age, period, or cohort compared to the overall average rate. Analysis was implemented by APCG1 package in R language (19).

## Results

### Prevalence of Overweight and Obesity

Overall, the percentages of boys and girls were 52.32 and 47.68%, respectively. Boys and girls had similar age, grade, and period percentages (Table 2). Boys had a significantly greater height (138.37 vs. 137.83 cm, *p* < 0.001), weight (37.18 vs. 35.59 kg, *p* < 0.001), and BMI (18.64 vs. 18.07, *p* < 0.001) compared to girls. The percentages of overweight (13.75 vs. 11.72%) and obesity (15.98 vs. 12.31%) were found higher in boys than in girls (*p* < 0.001). Our data showed that the prevalence of overweight declined from 13.17% in 2009 to 12.63% in 2018. In contrast, the prevalence of obesity increased from 13.19% in 2009 to 14.97% in 2018.

**Table 2:** Sample characteristics among boys and girls.

**Characteristics**	**Boys (*n* = 49,522)**	**Girls (*n* = 45,139)**	***p*-value**
Age (yrs)	9.96 ± 2.47	9.98 ± 2.46	0.063
Grade, *n* (%)			0.058
1	14,288 (28.85)	12,709 (28.15)	
4	15,990 (32.29)	14,752 (32.68)	
7	19,244 (38.86)	17,678 (39.16)	
Height (cm)	138.37 ± 16.77	137.83 ± 15.96	<0.001
Weight (kg)	37.18 ± 15.13	35.59 ± 13.42	<0.001
BMI	18.64 ± 4.10	18.07 ± 3.70	<0.001
BMI group, *n* (%)			<0.001
Underweight	3,043 (6.14)	2,988 (6.62)	
Normal	31,755 (64.12)	31305 (69.35)	
Overweight	6,812 (13.75)	5290 (11.72)	
Obese	7,912 (15.98)	5556 (12.31)	
Living area, *n* (%)			0.017
Industrial area	31,365 (63.33)	28,320 (62.74)	
Semi-industrial area	3,211 (6.48)	3,123 (6.92)	
Remote area	14,950 (30.19)	13,697 (30.34)	
Period, *n* (%)			0.730
2009	5,714 (47.86)	6,226 (52.14)	
2010	5,266 (47.57)	5,805 (52.43)	
2011	4,938 (48.19)	5,310 51.81)	
2012	4,775 (47.37)	5,306 (52.63)	
2013	4,520 (47.71)	4,953 (52.29)	
2014	4,329 (48.30)	4,633 (51.70)	
2015	4,067 (46.88)	4,608 (53.12)	
2016	3,890 (47.56)	4,289 (52.44)	
2017	3,822 (48.03)	4,136 (51.97)	
2018	3,818 (47.29)	4,256 (52.71)	

**Figure 1:**
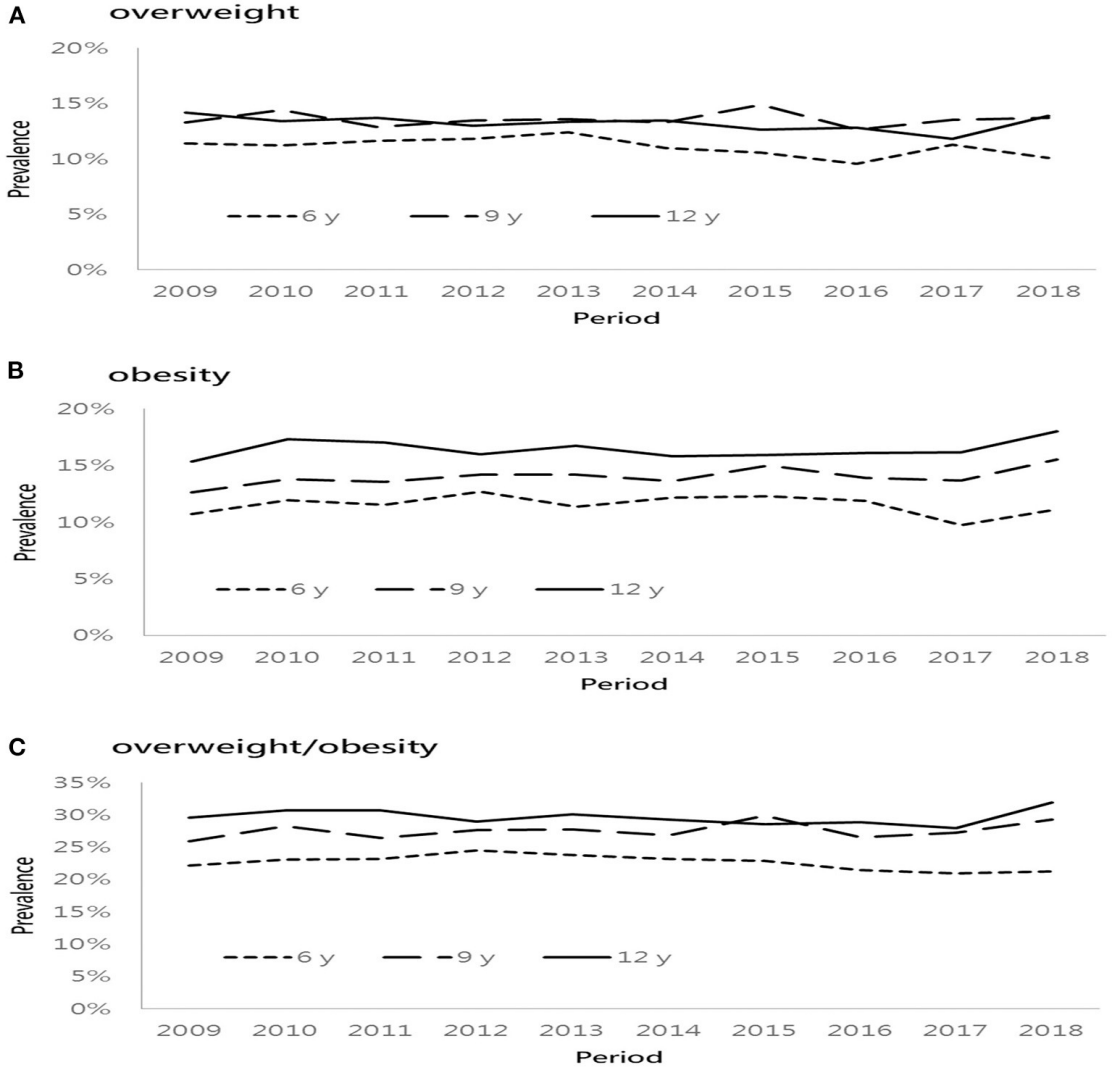
Age-specific prevalence of overweight and obese from 2009 to 2018. **(A)** overweight, **(B)** obesity, and **(C)** overweight/obesity.

**Figure 2:**
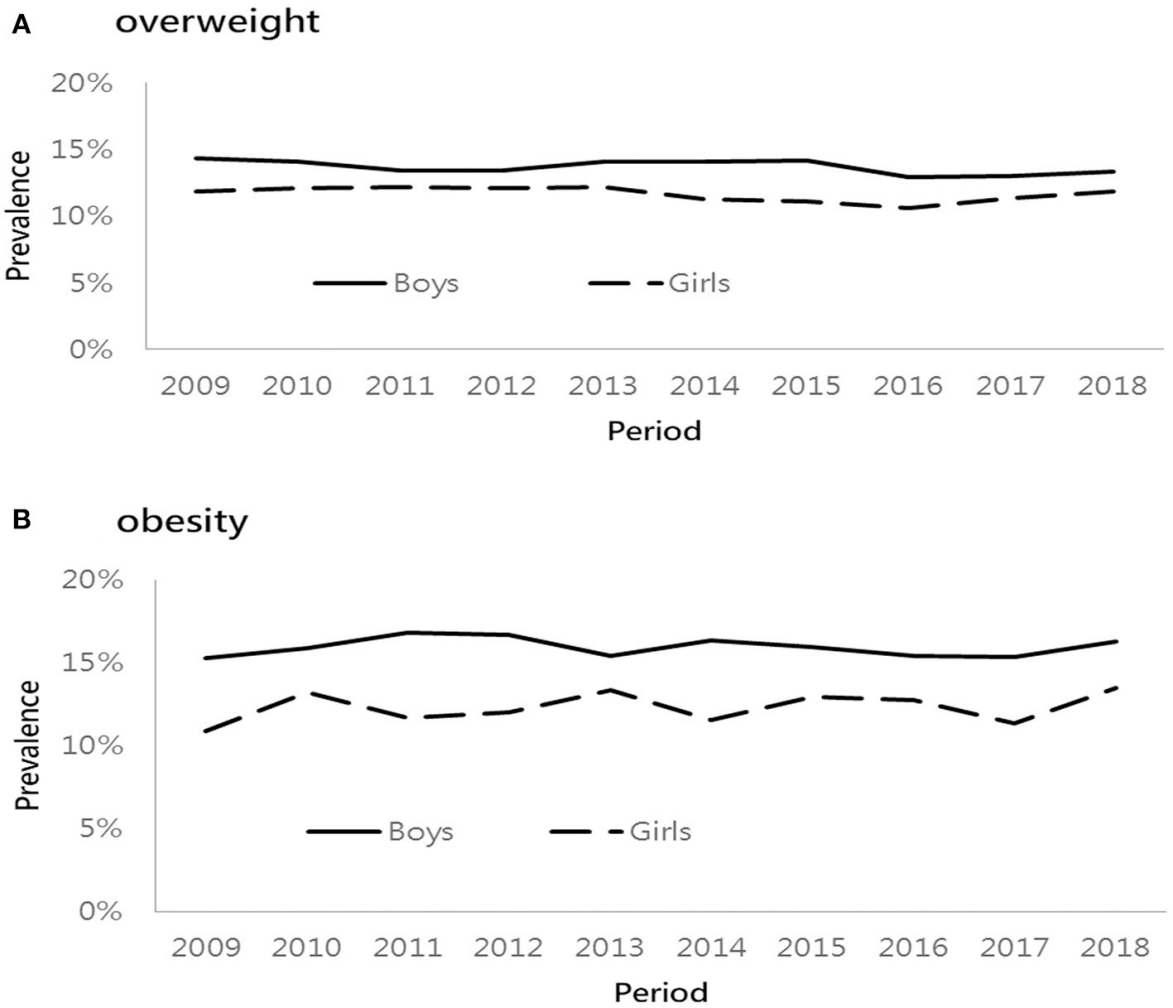
Sex-specific prevalence of overweight and obese from 2009 to 2018. **(A)** overweight and **(B)** obesity.

Figure 1 presents the prevalence of overweight and obesity based on three age groups from 2009 to 2018. The lowest percentage of overweight was found at the age of 6 years, followed by similar prevalence rates at the age of 9 and 12 years. However, the percentage of obesity increased with age regardless of time period. In general, the prevalence of overweight/obesity was higher in the older age group except that in 2015. Among different age groups of both boys and girls, the prevalence of overweight and obesity increased progressively, especially in children aged 9 and 12 years, respectively. Figure 2 presents the sex difference of the prevalence of overweight and obesity. From 2009 to 2018, boys had a higher prevalence of overweight than girls, as well as a trend of obesity. In both genders, the prevalence of overweight declined in 2016. Additionally, the prevalence of obesity decreased in 2017. It is worthy of mentioning that the time trend of obesity in girls was more fluctuating than that in boys.

Table 3 lists the prevalence of overweight and obesity across six birth cohorts and periods in both genders. Across three age groups, the prevalence of overweight increased at the age of 9 years and then became stable until 12 years of age for each cohort. For instance, the prevalence of overweight among the boys, evaluated in 2016–2018, was higher in boys born in 2007–2009, compared with those born in 2010–2012. The prevalence of obesity increased by age for each cohort. For example, boys born in 2004–2006 had the highest prevalence of obesity (18.68%), evaluated in 2016–2018, followed by those born in 2007–2009 (15.5%) and 2010–2012 (12.29%). Across different birth cohorts, boys who were born in the recent cohort (2004–2006 or 2007–2009) had a higher percentage of obesity than those born in the earlier cohort. A similar pattern was also observed among girls.

**Table 3:** Prevalence (%) of overweight and obesity by period and birth cohort, 2009–2018.

	**Birth cohort**	**Period**							
		**2009**		**2010–2012**		**2013–2015**		**2016–2018**	
		**Overweight**	**Obesity**	**Overweight**	**Obesity**	**Overweight**	**Obesity**	**Overweight**	**Obesity**
**Boys**									
	1997	14.92	18.90						
	1998–2000	14.52	13.78	13.93	19.28				
	2001–2003	13.28	12.09	14.41	15.57	14.00	17.60		
	2004–2006			12.52	13.61	14.67	15.24	13.55	18.68
	2007–2009					12.99	13.43	14.41	15.50
	2010–2012							11.24	12.29
**Girls**									
	1997	13.45	11.54						
	1998–2000	12.12	11.46	12.77	14.12				
	2001–2003	9.25	9.12	12.80	11.95	11.07	13.07		
	2004–2006			10.47	10.30	13.09	13.19	12.11	14.57
	2007–2009					9.56	10.32	12.13	13.18
	2010–2012							9.30	9.39

### Age, Period, and Cohort Effect

**Figure 3:**
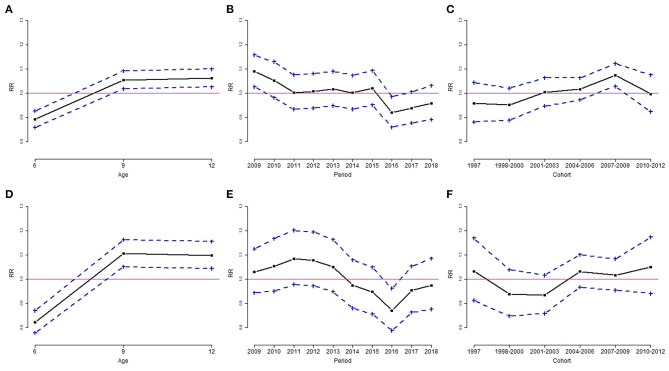
Age, period, and cohort effect of the rate ratio (RR) of overweight by sex group, for boys **(A–C)** and girls **(D–F)**. **(A,D)**: age effect; **(B,E)**: period effect; **(C,F)**: cohort effect. Results are RR (solid line) and 95% confidence interval (dotted line). The solid horizontal line represents RR = 1.

**Figure 4:**
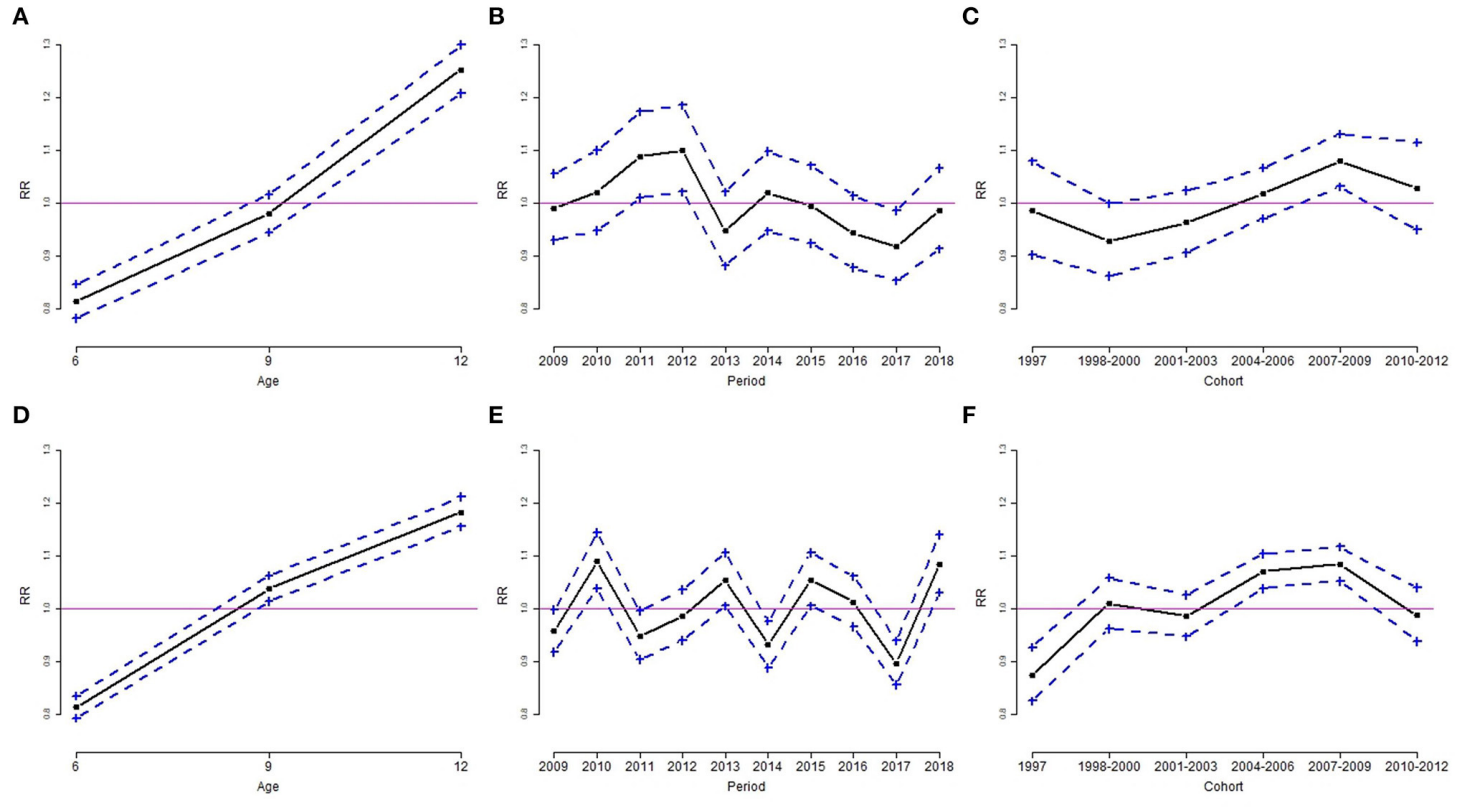
Age, period, and cohort effect of the rate ratio (RR) of obesity by sex group, for boys **(A–C)** and girls **(D–F)**. **(A,D)**: age effect; **(B,E)**: period effect; **(C,F)**: cohort effect. Results are RR (solid line) and 95% confidence interval (dotted line). The solid horizontal line represents RR = 1.

Results from the Poisson log-linear model using the intrinsic estimator method are shown in Table 4. The RR and 95% confidence interval of overweight and obesity are depicted in Figures 3, 4. The age effect was significantly associated with the prevalence of overweight (Figures 3A,D) and obesity (Figures 4A,D) in both genders. Children at the age of 6 years had a significant lower risk of overweight and obesity in both genders. Boys aged 9 years had a higher risk of overweight, but the risk of obesity was not significant at the age of 12. Among girls, the elevated risk of overweight and obesity was seen at the age of 9 and 12 years.

**Table 4:** Age period cohort analysis of children overweight and obesity by sex group, 2009–2018.

		**Overweight**				**Obesity**			
		**Boys**		**Girls**		**Boys**		**Girls**	
		**Coefficient**	**SE**	**Coefficient**	**SE**	**Coefficient**	**SE**	**Coefficient**	**SE**
Age	6	−0.114*	0.02	−0.195*	0.03	−0.206*	0.02	−0.206*	0.01
	9	0.053*	0.02	0.101*	0.03	−0.020	0.02	0.038*	0.01
	12	0.060*	0.02	0.094*	0.03	0.226*	0.02	0.169*	0.01
Period	2009	0.087*	0.03	0.030	0.04	−0.009	0.03	−0.043	0.02
	2010	0.052	0.04	0.052	0.05	0.021	0.04	0.087*	0.02
	2011	0.003	0.04	0.081	0.05	0.086*	0.04	−0.052	0.02
	2012	0.008	0.04	0.075	0.05	0.096*	0.04	−0.013	0.02
	2013	0.017	0.04	0.050	0.05	−0.052	0.04	0.053*	0.02
	2014	0.002	0.04	−0.025	0.05	0.020	0.04	−0.071*	0.02
	2015	0.020	0.04	−0.053	0.05	−0.005	0.04	0.054*	0.02
	2016	−0.083*	0.03	−0.138*	0.05	−0.058	0.04	0.013	0.02
	2017	−0.063	0.03	−0.047	0.05	−0.086*	0.04	−0.109*	0.02
	2018	−0.042	0.04	−0.025	0.05	−0.013	0.04	0.081*	0.03
Birth cohort	1997	−0.041	0.04	0.033	0.06	−0.013	0.05	−0.134*	0.03
	1998–2000	−0.049	0.04	−0.063	0.05	−0.074	0.04	0.010	0.02
	2001–2003	0.004	0.03	−0.068	0.04	−0.037	0.03	−0.014	0.02
	2004–2006	0.017	0.02	0.032	0.03	0.018	0.02	0.069*	0.02
	2007–2009	0.072*	0.02	0.017	0.03	0.077*	0.02	0.081*	0.02
	2010–2012	−0.003	0.04	0.050	0.06	0.029	0.04	−0.012	0.03

Bold values with * are significant at p-value < 0.05.

The period effect of overweight was similar for boys and girls. The downward trends of overweight in both genders occurred in 2016 (Figures 3B,E). The estimated coefficient of period effect of overweight in 2016 was −0.083 (RR = 0.92, *p* < 0.05) and −0.138 (RR = 0.87, *p* < 0.05) for boys and girls, respectively (Table 4). In addition, boys had a higher risk of overweight in 2009 compared to the overall rate of overweight. The trends of obesity were quite different in both genders. Boys had a significant greater risk of obesity from 2011 to 2012 and a lower risk of obesity in 2017 (Figure 4B). The estimated coefficient of period effect of obesity in 2017 was −0.086 (RR = 0.92, *p* < 0.05) for boys. There was a greater fluctuation in period effect of obesity among girls. Higher RR of obesity in girls was found in 2010, 2013, 2015, and 2018. The risks of obesity in 2014 (coefficient = −0.071, RR = 0.93, *p* < 0.05) and 2017 (coefficient = −0.109, RR = 0.90, *p* < 0.05) were significantly lower than the average rate of obesity in girls (Table 4; Figure 4E).

With regard to birth cohort effects, results indicated that recent cohorts had a significantly higher risk of overweight or obesity in both genders. Boys from birth cohorts of 2007 to 2009 had a significantly higher risk of overweight and obesity (Figures 3C, 4C). Girls from birth cohorts of 2004 to 2009 had significantly greater risk of obesity. Interestingly, girls who were born in 1997 had a significant low risk of obesity (Figure 4F).

## Discussion

This is the first study to examine the APC effects in childhood overweight and obesity in Taiwan. Using data collected from schoolchildren between 2009 and 2018, our results from the APC analysis showed a significant positive age effect to the rising overweight and obesity trends. The age effect to overweight and obesity among children aged 6 years in both genders was significantly lower than that of the overall groups. In contrast, the age effect to overweight and obesity among children aged 12 years was significantly higher than that of the overall groups in our study. Among children aged 9 years for both boys and girls, the age effect also had a significant contribution to overweight and obesity (Figures 3A,D, 4D), except for the effect to obesity in boys (Figure 4A). This finding is similar to other reports that the prevalence of overweight and obesity increased gradually with increasing age (5, 10, 20). Pubertal physical growth including height and weight contributed to the progressively rising BMI in this age group.

We proposed the difference of age effect in this age group between boys and girls, possibly due to the different timing of pubertal growth spurt, which is 10–12 years of age in girls and 12–14 years of age in boys. Aris et al. demonstrated that the associations with greater standing height and trunk length and earlier pubertal development in adolescence were more pronounced for BMI measures in later childhood (21). Also, children with higher BMI during infancy and childhood demonstrated faster subsequent growth in stature, for both standing height and trunk length. Additionally, children with higher BMI showed earlier pubertal development and slower linear growth during adolescence. Thus, they speculated that factors known to influence linear growth and positively correlate with BMI, such as insulin-like growth factor 1 (IGF-1) and fasting insulin, may play an important role in the relationship between higher BMI in early life and accelerate subsequent linear growth. The regulation of the growth hormone (GH)–IGF-1 axis has also been associated with prepuberty BMI (22). Rafael et al. illustrated that muscle mass increased greatly in boys as did fat mass in girls within the pubertal period. It has been hypothesized that part of these changes can be justified by the action of sex hormones, which begin to rise during puberty (23).

For period effect in our APC analysis, the risk of overweight in 2016 and obesity in 2017 declined significantly among both boys (overweight: RR = 0.92; obesity: RR = 0.92) and girls (overweight: RR = 0.87; obesity: RR = 0.90) within this 10-year study period (Table 4; Figures 3B,E, 4B,E). The decreasing trend with period is not consistent with previous studies (8–10) other than that in Taiwan (11). We speculated two possible reasons for the declining trend. First, the Ministry of Health and Welfare in Taiwan launched the national weight-loss campaign since 2011, and the Hualien County Health Bureau strongly reinforced the awareness and strategy of better health to support weight-loss campaigns in 2016. Second, the school nutrition policy “Beverage and snack selling rule in school, 2016” by the Ministry of Education in Taiwan since 2016/11/21 banned the soft drink in the senior high school campus, and only the following commercial items could be sold in the elementary and junior high school campuses: 100% fruit or vegetable juice, plain water, fresh milk, extended shelf-life milk, soymilk, yogurt, etc., which should reduce the opportunity to purchase beverage and high-calorie snack at daytime in school. Although in 2018 the rebounded prevalence of overweight and obese did not reach a significant level, the obesogenic environment such as unhealthy high-energy food and daily sedentary behavior should be transformed to support healthful decision and prevent the progression of childhood obesity (24–26).

As for cohort effect in our APC analysis, we demonstrated that later birth cohorts (2004–2006 and 2007–2009) had significant positive effects to increased risk of overweight and obesity among both boys (Figures 3C, 4C) and girls (Figure 4F). This is comparable with the findings of previous reports (8, 9, 11). Chu et al. represented the national population-based study related to APC analysis for Taiwanese adolescent aged 12–18 years between 2006 and 2014 (11). They concluded the birth cohorts of the 1990s: the younger cohorts had greater odds of being overweight and obese than the older cohorts when they reached adolescence. Birth cohort effects reflect early-life factors and environmental changes, such as maternal increased food consumption and sedentary behaviors, or during the economic transition (10). Children born between 2004 and 2009 (i.e., generation z) grew up in a convenient environment fulfilled with high technologic services; thus, they are currently exposed to obesogenic environments. Moreover, further studies should be conducted to examine the causal relationship of positive birth cohort effects in relation to child obesity.

Ogden et al. reported that the percentage of obese children in the United States aged 6 to 11 increased from 7% in 1980 to 18% in 2012, and at the same time, the percentage of obese teenagers aged 12–19 increased from 5 to 21% which could represent the influence of a convenient environment to children and adolescents in generation z (27). An obesogenic environment could be modified if teachers and parents worked together to build up healthy food intake and physical activity habits. They illustrated that healthy dietary habits (eating breakfast at home, bringing a school lunch, and not bringing money to purchase food) had a lower risk of obesity. The quality of the eaten food was associated with a risk of obesity: fruit was inversely associated with the risk of obesity; on the contrary, sweetened beverages and refined carbohydrates with added fat were associated with increased risk of obesity (26). Hsiao et al. (28) mentioned that to achieve good family health, physicians should engage the concerns of parents to empower their knowledge of healthy literacy in nutrition, exercise, and parenting skills. A family-based exercise plan, healthy diet plan, and structural daily schedule will provide a supportive environment for children after leaving the school campus. Reward options should not include snacks, sweetened beverages, or high-calorie foods; instead, outdoor sports or tours are recommended to promote physical activity. Management of childhood obesity is progressive and age-dependent, and it should be initiated as early as possible (29, 30).

Based on the report of the Commission on Ending Childhood Obesity by the World Health Organization (WHO) in 2016, their recommendations were to promote the following: intake of healthy food; physical activity; preconception and pregnancy care; early childhood diet and physical activity; health, nutrition, and physical activity for school-aged children; and weight management. Comprehensive programs that promote healthy school environments, health and nutrition literacy, and physical activity among school-age children and adolescents should be established and assisted by family-based, multicomponent lifestyle weight management services (31). In 2018, the same commission by WHO illustrated another action report on childhood obesity and set global targets for halting the increase in obesity, including no increase in overweight among children under age 5, school-age children, or adolescents by 2025 (from 2010 levels). An action to reverse the epidemic is the focus of the recommendations made by the WHO Commission on Ending Childhood Obesity and is one of the main objectives of the Decade of Action on Nutrition (32). The collaboration between school, family, and community is crucial to establishing supportive infrastructure for ending childhood obesity especially in school-age children.

According to our results, we proposed that the timing for weight intervention of schoolchildren could be launched as early as the first grade of elementary school. The outdoor recess between lessons in school should be emphasized to increasing physical activity. “Emptying the classroom at recess” and “Zero hour PE” are good options for school-age children to become mobile in their daily schedule. A recent report (33) concluded that school- and community-based physical activity interventions as part of an obesity prevention or treatment program can benefit executive functions of children with obesity or overweight specifically. Similarly, school-based dietary interventions may benefit general school achievement in children with obesity. Cuda et al. illustrated an algorithm that breaks down intake guidelines for age groups between infancy and adulthood. In considering how to modify the food intake of a child with obesity, there is no universally accepted approach. An understanding of an appropriate intake for a normal-weight child is necessary as a starting point. Children with obesity should not be given any sugar-sweetened beverage or any fast food or desserts (29). School authority should collaborate with shops and restaurants nearby the campus to provide healthy food options for breakfasts and afternoon snacks based on the school nutrition policy “Beverage and snack selling rule in school, 2016” by the Ministry of Education in Taiwan. Among the overweight and obese children detected by the regular health checkup, the notification letter should be given to their parents and enhance their awareness of the consequence of childhood obesity.

The strength of this study is that we used a total of 94,661 schoolchildren recruited from 13 townships in Hualien county between 2009 and 2018. Three age groups of the participants included the first (aged 6 years), fourth (aged 9 years), and seventh grades (aged 12 years). The composition of our dataset showed no significant difference in terms of annual subject number and ratio of boys vs. girls; therefore, our dataset could represent this target age population. Furthermore, body height and weight were actual data measured individually by trained staffs, other than self-reported, which could minimize recall bias. There were some limitations in this study that need to be addressed. First, metabolic status and daily physical activity, dietary records, and anthropometrics such as previous BMI status and waist circumference were not available in the annual health examination dataset. In the future, we should try to collect these data to analyze the effects of these factors on overweight and obesity among schoolchildren. Second is the discontinuous age, e.g., 6, 9, and 12 years, in our dataset, which limits the understanding of age effects with continuous aspects. Third, our data may only illustrate the trend among the schoolchildren of Hualien county, not in other regions of Taiwan.

## Conclusions

In summary, our study showed a significant association of age, period, and cohort effects on the risk of overweight and obesity among school-age children. With increasing age, regardless of fluctuations, the trend in the prevalence of overweight and obesity increased in both genders. For the period effect, the trend in the prevalence of overweight and obesity fluctuated downward slowly from 2016 to 2017 in boys and girls. For cohort effects in both genders, the birth cohort of 2007 to 2009 was correlated with the increased risk of overweight and obesity.

## Data Availability Statement

The datasets presented in this article are not readily available because the datasets used in this study will not be made publicly available. The Ethics committee did not give permission for the data to be made publicly available. Requests to access the datasets should be directed to Y-CC, tonyijane@hotmail.com.

## Ethics Statement

The studies involving human participants were reviewed and approved by Hualien Tzu Chi Hospital. Written informed consent to participate in this study was provided by the participants' legal guardian/next of kin.

## Author Contributions

Y-CC and S-HW: study design and coordination of data collection and manuscript writing/editing. S-HW: statistical analysis. W-HH, S-FH, and HH: data interpretation and revision of the manuscript. Y-WW and C-HC: critical review of the manuscript. All authors contributed to the article and approved the submitted version.

## Conflict of Interest

The authors declare that the research was conducted in the absence of any commercial or financial relationships that could be construed as a potential conflict of interest.
