# Supplementary material for: Sex-Specific Temporal Trends in Overweight and Obese Among Schoolchildren From 2009 to 2018: An Age Period Cohort Analysis
Source: Front Pediatr. 2021 May 13;9:615483. doi: 10.3389/fped.2021.615483 (PMC8155382; doi:10.3389/fped.2021.615483)
Supplement: Supplementary file 1 [file Data_Sheet_1.PDF]

Supplementary Table. Birth cohort structure of 6 synthetic birth cohorts by intention to collapse method of the study data, 2009-2018.

synthetic periods and ages	2009	2010-2012	2013-2015	2016-2018
6 y	C3 (2003)	C4 (2004-2006)	C5 (2007-2009)	C6 (2010-2012)
9 y	C2 (2000)	C3 (2001-2003)	C4 (2004-2006)	C5 (2007-2009)
12 y	C1 (1997)	C2 (1998-2000)	C3 (2001-2003)	C4 (2004-2006)
